# UCS Protein Rng3p Is Essential for Myosin-II Motor Activity during Cytokinesis in Fission Yeast

**DOI:** 10.1371/journal.pone.0079593

**Published:** 2013-11-14

**Authors:** Benjamin C. Stark, Michael L. James, Luther W. Pollard, Vladimir Sirotkin, Matthew Lord

**Affiliations:** 1 Department of Molecular Physiology and Biophysics, University of Vermont, Burlington, Vermont, United States of America; 2 Department of Cell and Developmental Biology, State University of New York - Upstate Medical University, Syracuse, New York, United States of America; Cancer Research UK London Research Institute, United Kingdom

## Abstract

UCS proteins have been proposed to operate as co-chaperones that work with Hsp90 in the *de novo* folding of myosin motors. The fission yeast UCS protein Rng3p is essential for actomyosin ring assembly and cytokinesis. Here we investigated the role of Rng3p in fission yeast myosin-II (Myo2p) motor activity. Myo2p isolated from an arrested *rng3-65* mutant was capable of binding actin, yet lacked stability and activity based on its expression levels and inactivity in ATPase and actin filament gliding assays. Myo2p isolated from a *myo2-E1* mutant (a mutant hyper-sensitive to perturbation of Rng3p function) showed similar behavior in the same assays and exhibited an altered motor conformation based on limited proteolysis experiments. We propose that Rng3p is not required for the folding of motors *per se*, but instead works to ensure the activity of intrinsically unstable myosin-II motors. Rng3p is specific to conventional myosin-II and the actomyosin ring, and is not required for unconventional myosin motor function at other actin structures. However, artificial destabilization of myosin-I motors at endocytic actin patches (using a *myo1-E1* mutant) led to recruitment of Rng3p to patches. Thus, while Rng3p is specific to myosin-II, UCS proteins are adaptable and can respond to changes in the stability of other myosin motors.

## Introduction

**Figure 1:**
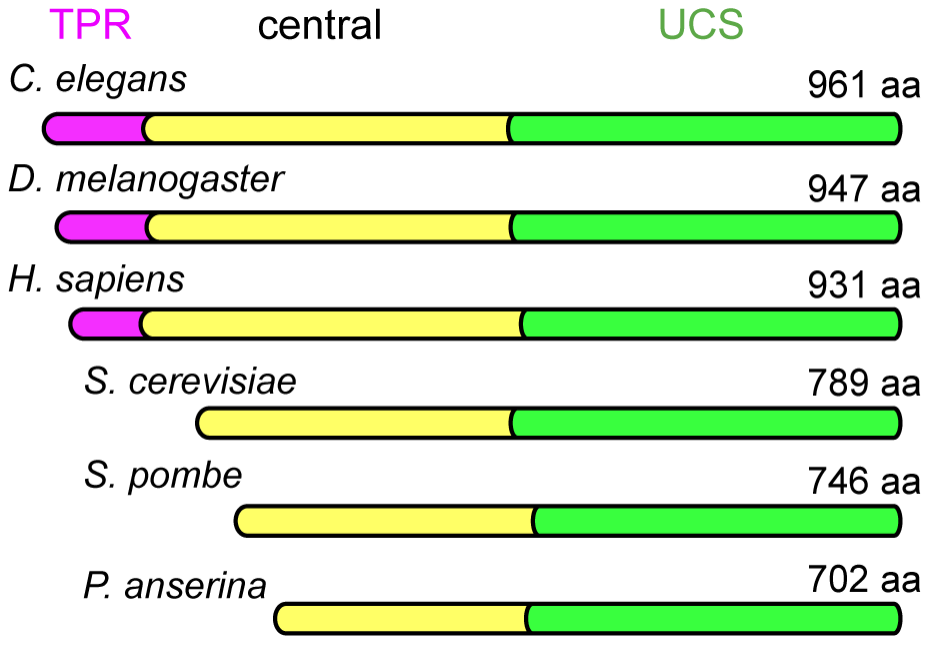
Schematic showing relative size and domain organization of a sample of UCS proteins. UCS proteins are a highly conserved family of proteins by virtue of the C-terminal myosin motor-binding UCS domain (green). Higher eukaryotes have a TPR domain (pink) at the N-terminus, whereas fungal homologs (e.g. *S. cerevisiae* She4p, *S. pombe* Rng3p, and *P. anserine* Cro1p) lack this TPR domain. The central domain (yellow) is the most variable domain between family members. The *H. sapiens* UCS protein included here is the striated muscle isoform.

Unc-45/Cro1/She4 (UCS) proteins are a family of myosin motor regulators that are conserved across eukaryotes [1]. UCS proteins typically contain a C-terminal UCS domain that binds myosin motors, a variable central domain that may play a role in oligomerization [2], and an N-terminal tetratricopeptide repeat (TPR) domain that interacts with the Hsp90 chaperone (Figure 1) [3]. Interaction between the UCS protein and Hsp90 has been shown to be important for the latter stages of the *de novo* folding of muscle myosin-II motors [4], [5]. Here the UCS protein may act as a co-chaperone that accelerates motor folding by bringing motors bound at the UCS domain into close contact with Hsp90 bound by the TPR domain [1], [2], [4].

Defects in UCS protein function have been associated with various diseases including arteriovenous malformation, cardiomyopathy, and cancer [6]–[9]. Studies from *C. elegans* have shown that misregulated levels of UCS protein (Unc-45) expression result in improper myofibril organization in body wall muscle, where either lack or over-expression of Unc-45 results in a decrease in thick filament assembly and paralysis [10], [11]. Similar defects are seen when UCS protein function is attenuated in zebrafish and *Drosophila* [9], [12]–[14].

Interestingly, fungal UCS proteins lack a TPR domain (Figure 1), suggesting an alternative role for the UCS family beyond acting as co-chaperones for Hsp90. Any such role is unlikely unique to fungi given that *C. elegans* Unc-45 does not depend on its TPR domain for function in the cell [15]. An Hsp90-independent role may well define the major role of UCS proteins in the regulation of myosin. Indeed, Hsp90 was recently proposed to be inhibitory to *C. elegans* Unc-45 as the two proteins compete for binding to myosin-II [15]. Furthermore, UCS proteins alone can limit myosin motor aggregation in vitro [3], [16], [17] consistent with an Hsp90-independent role in motor stabilization.

Fission yeast (*Schizosaccharomyces pombe*) provides a versatile and tractable model to study myosin-II and its role in actomyosin ring function and cytokinesis [18], [19]. Myo2p motor activity is required to drive contractile ring assembly following the accumulation of ring pre-cursors (nodes) at the medial division site in early mitosis [20], [21]. Previous work on the fission yeast UCS protein (Rng3p) has shown that it is essential for actomyosin ring formation [22] and is capable of enhancing the in vitro motility and apparent actin affinity of purified Myo2p [23], [24]. Use of different mutant alleles has shown that Rng3p works in the same genetic pathway as fission yeast Myo2p and Hsp90 [22], [25], suggesting a role in Myo2p folding or stabilization.

Studies from budding yeast (*Saccharomyces cerevisiae*) and fission yeast have shown that the UCS proteins from both yeasts associate with all the myosin motors found in their respective system [26], [27]. Nevertheless the budding yeast UCS protein is only required for the function of three of the five myosins: the type-I myosins Myo3p and Myo5p and the type-V myosin Myo4p [27], [28]. While previous studies have implicated Rng3p in Myo2p function [22]–[24], it is not clear whether Rng3p is required for the function of any of the other fission yeast myosins.

In this study we use a combination of genetics, cell biology, and biochemical studies to examine the molecular mechanism by which Rng3p regulates myosin motor function in fission yeast. We show that Rng3p is specific to myosin-II and the contractile ring and is essential for Myo2p motor activity. Nevertheless, mutational perturbation of myosin-I motor function led to recruitment of Rng3p to myosin-I patches in vivo. Thus, UCS proteins appear to have some flexibility with regard to their substrate-specificity which may facilitate functional adaptation of myosin motor regulation in response to changes in stress or cellular environment.

## Materials and Methods

### Yeast Strains and Plasmids

Yeast strains used in this study are listed in Table S1. All strains were constructed by genomic integrations using homologous recombination or back-crossing following established protocols [29], [30]. The *myo1-E1* strain was constructed by introducing the G308R mutation into the 5 kb EcoR1-fragment of the *myo1* gene (containing 619 nucleotides of 5′ UTR, ORF, and 646 nucleotides of 3′ UTR) in pBluescript [31] by PCR-based mutagenesis. The mutagenized 5 kb EcoR1 fragment was transformed into a *myo1*Δ*ura4^+^* strain, followed by 5-FOA selection to isolate transformants which integrated the mutated *myo1-E1* sequence in place of the *ura4^+^* marker. Isolated transformants were verified by PCR and sequencing.

Plasmids pGST-*LEU2-cdc4*, pGST-*LEU2-rlc1*, and pGST-*ura4-rlc1* [23] were employed to facilitate purification of Myo2p samples. The pMyo2p-S1-GFP plasmid was used to over-express a C-terminal GFP-tagged sub-fragment 1 form of Myo2p (motor plus light chain-binding domains: amino acids 1–815, bp 1–2445). This plasmid was generated by transferring the *Not*I/*Not*I *myo2* fragment from pDS472a-myo2-head [24] into pDS572a. The pMyo2p-S1-FLAG plasmid was used to over-express a C-terminal FLAG-tagged S1 form of Myo2p. This plasmid was generated by first amplifying a 3′ portion of the S1 coding region (bp 2128–2445) from pDS472a-myo2-head using a 5′ *myo2* primer (centered on a native *Kpn*I site: CCAACA*GGTACC*TATGTGGAATC) and a 3′ primer in which DNA encoding the FLAG tag (underlined) and a stop codon was included upstream of the *Not*I cloning site at the 3′ end of the *myo2* S1 sequence (5′ *Sal*I ACGC*GTCGAC*TTACTTATCGTCATCGTCTTTGTAGTCGCTTCC *GCGGCCGC*CGGGCCTTAGAT). The *Kpn*I/*Sal*I S1-FLAG fragment was ligated into pDS472a-myo2-head in place of the *myo2-GST Kpn*I/*Sal*I fragment. pDS573a, pDS573a-*LEU2*, pYFP*-myo2*, pGST*-rng3-FL*, and pGFP*-rng3* plasmids [23], [24], [32] were also employed in this study as control and test plasmids when examining the impact of Rng3p and Myo2p over-expression (Figure S2).

Site-directed mutagenesis by overlap extension [33] was performed on the *myo2* S1 sequence to incorporate the -E1 mutation (G345R). Flanking primers utilized the native *Nhe*I (bp 571) and *Kpn*I (bp 2128) sites within the *myo2* S1 sequence (5′ *Nhe*I-*myo2*: CT-TGCAGCAATT*GCTAGC*TCG; 3′ *Kpn*I-*myo2*: GATTCCACATA*GGTACC*TGTTGG). The *myo2-E1* mutagenesis primers employed the forward (and reverse) of bp 1024–1052 of the *myo2* sequence incorporating a Gly to Arg amino acid substitution (GGA to CGA, underlined): CTACATATGCGAAATATTGATGTCGGTGC. The mutant *Nhe*I/*Kpn*I fragment was transferred into pDS472a-myo2-head and subsequently modified (to generate GFP- and FLAG-tagged constructs) as described above. The fidelity of all constructs was confirmed by DNA sequencing.

The *LEU2* GFP-*myp2*-head/*myo2*-tail chimera plasmid [34], along with empty vector and GFP-*rng3* control plasmids [24] were used to test whether the Myp2p motor relied on Rng3p function. The chimera and control constructs were transformed and expressed in temperature-sensitive *rng3-65* cells grown at the permissive temperature (25°C) followed by a shift to the restrictive temperature (36°C) to test their ability to rescue function and cell growth.

### Microscopy

Myo1p-GFP patch dynamics were tracked using time-lapse imaging of cells. Cells were mounted on a flat media pad (30 µl +1% agarose) on a slide surface and a coverslip was then sealed above with a 1∶1∶1 ratio of petroleum jelly, lanolin, and paraffin. Each frame consisted of a z-stack (0.3 µm for seven sections) collected on an Applied Precision Delta Vision imaging station constructed on an Olympus IX-70 inverted microscope base with a 1006 oil immersion lens (1.4 NA) and immersion oil with a refractive index of 1.518. Images were deconvolved using the point-spread functions and software supplied by the manufacturer. Maximum intensity projections of the deconvolved 3D stacks were then used to measure patch lifetimes. Subsequent analysis employed ImageJ (http://rsbweb.nih.gov/ij/), Microsoft Excel (Redmond, WA), and GraphPad Prism software (La Jolla, CA).

Myo52p-3xGFP, Rng3p-3xGFP, Cam2p-^m^Cherry, Myo1p-GFP (single plane), and Sad1p-CFP localization was imaged in cells using a Nikon TE2000-E2 inverted microscope. A motorized fluorescence filter turret and a Plan Apo 60X (1.45 NA) objective was used to capture DIC and epifluorescence cell images (Melville, NY) with an EXFO X-CITE 120 illuminator (Nikon). NIS elements software (Nikon) was used to control the microscope, two Uniblitz shutters (Vincent Associates, Rochester, NY), and the cameras. A Photometrics CoolSNAP HQ2 14-bit camera (Tucson, AZ) was employed for Rng3p-3xGFP, Cam2p-^m^Cherry, and Myo1p-GFP (single planes) and Sad1p-CFP (z-stack, 0.3 µm for seven sections), and an Andor iXon 897 EMCCD camera (Belfast, Northern Ireland) was used for time-lapse tracking of Myo52p-3xGFP particles (single planes). Cells were mounted as described above.

### Calculating Doubling Time

Cells grown in YE5S media at 25°C until saturation were back-diluted to an OD_595 nm_ of 0.035 in fresh YE5S media. Cells were then allowed to continue to grow at 25°C with cell density measured every 60–90 minutes until an OD_595 nm_ of 1.4 or greater was reached. Calculation of the doubling time was done in GraphPad Prism using the linear (exponential) phase of the growth curve.

### Purification of Motors

Full-length and S1 forms of Myo2p (and -E1) plus light chains were purified as previously described [23], [24]. For motility assays with S1 forms, S1-GFP fusions were employed following one-step purification on glutathione Sepharose (GE Healthcare) via the GST-tagged light chains. S1-FLAG forms were purified in two steps via the GST-tagged light chains and by a second step on anti-FLAG resin (Sigma).

Rng3p is essential for *S. pombe* growth, and as such cannot be deleted from the genome. We therefore performed isolations of full-length Myo2p in temperature shift assays to assay its dependence on Rng3p function. One-liter cultures of wild-type and temperature-sensitive *rng3-65* cells were induced to over-express Myo2p heavy and light chains grown at the permissive (25°C) temperature for 16 hours. The cultures were then split evenly into two 500 ml cultures and then brought to a final volume of one liter using temperature-equilibrated media, one remained at 25°C and the other was grown at the restrictive temperature (37°C) to eliminate Rng3p function. The cells were then harvested 12 hours post-shift and Myo2p 1-step purified via GST-tagged light chains. Control samples in which Rng3p function was attenuated following the initiation of Myo2p over-expression were also performed in which cultures were split and shifted 22 hours post-induction [24].

### *In vitro* Motility Assays

Motility assays were based on an established protocol [35] with ∼40 µg/ml of myosin applied to the motility chambers. Fluorescent actin stocks were prepared at 5 µM from chicken skeletal muscle G-actin stocks purified from acetone powder as previously described [36] and polymerized with the addition of 50 mM KCl and 1 mM MgCl_2_ in the presence of 5 µM rhodamine phalloidin (Invitrogen) for 30 min. Motors were adhered to the surface of a nitrocellulose-coated coverslip for 10 min and the chamber was then washed as follows: (*a*) three times with motility buffer (25 mM imidazole pH 7.4, 50 mM KCl, 1 mM EGTA, 4 mM MgCl_2_, 2 mM DTT) plus 0.5 mg/ml BSA; (*b*) three times with motility buffer; (*c*) twice with motility buffer containing 1 µM vortexed (1 min) unlabeled actin filaments; (*d*) three times with motility buffer plus 1 mM ATP; (*e*) twice with motility buffer plus 25 nM rhodamine phalloidin-labeled actin filaments and oxygen scavengers (50 µg/ml catalase, 130 µg/ml glucose oxidase, and 3 mg/ml glucose); (*f*) twice with motility buffer plus 20 mM DTT, 0.5% methyl-cellulose and oxygen scavengers; and (*g*) twice with motility buffer plus 20 mM DTT, 0.5% methyl-cellulose, 1.5 mM ATP, and oxygen scavengers. S1-GFP fusions were indirectly attached to the motility surface using monoclonal GFP antibodies (50 µg/ml in motility buffer) [37]. Excess antibodies were removed from the nitrocellulose by the BSA wash (*a*) prior to 10 min incubation with the S1 samples and subsequent washes (*b–g*). Filaments were observed at room temperature and recorded by time-lapse imaging at 2 s intervals. ImageJ software with the MTrackJ plug-in was used to calculate average filament velocities.

### High-salt ATPase Assays

High-salt ATPase assays were carried out at room temperature in 2 mM Tris-HCl pH 7.2, 10 mM imidazole, 500 mM KCl, 2 mM ATP, 1 mM DTT and either 10 mM CaCl_2_ or 10 mM MgCl_2_ with 0.2–0.8 µg/ml myosin motors. Malachite green was used to quantitate P*_i_* release [38]. Background P*_i_* was subtracted from all values using controls lacking myosin. P*_i_* release was calculated using Microsoft Excel and GraphPad Prism software.

### Limited Proteolysis

Purified Myo2p and -E1 S1 samples were incubated with 200 ng Trypsin (Sigma) buffered in Tris-HCl pH 8.0 in a total reaction volume of 100 µl. For each time point, 20 µl was removed from the reaction and added to 8 µl 5X SDS loading buffer followed by boiling at 95°C for 5 min to stop proteolysis. After all time points had been taken, each sample was boiled again at 95°C for 5 min then loaded onto a 12% SDS-PAGE gel. The gel was then Coomassie-stained and scanned; densitometry analysis was performed using ImageJ. Data was analyzed using GraphPad Prism.

### Supporting Information

Figures S1 (Myo2p versus mutant Myo2-E1p ATPase data), S2 (Rng3p and Myo2p over-expression tests), S3 (Myo1p localization in wild-type versus *myo1-E1* cells), and Table S1 (fission yeast strains) can be found in this section. Movie S1 (motility activity of Myo2p purified from wild-type versus *rng3-65* cells), Movie S2 (motility activity of wild-type Myo2p S1 versus mutant Myo2-E1p S1 forms), Movie S3 (Myo1p patch dynamics in wild-type versus *rng3-65* cells), and Movie S4 (Myo52p particle movement in wild-type versus *rng3-65* cells) can also be found here.

## Results

### Myo2p Motor Activity Depends on Rng3p

**Figure 2:**
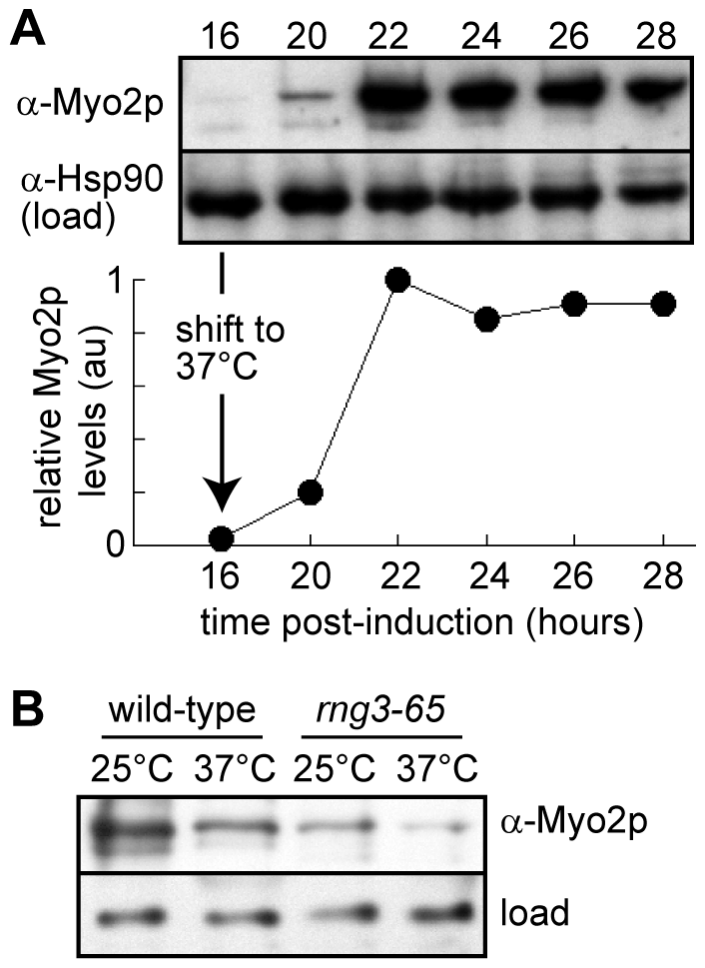
Isolating Myo2p in the absence of functional Rng3p. Full-length over-expressed Myo2p motors were purified from wild-type (*rng3^+^ nmt41^prom^-myo2*) and mutant (*rng3-65 nmt41^prom^-myo2*) cells following growth at the permissive (25°C) or restrictive (37°C) temperature. A) Western blot depicting Myo2p levels (upper panel) from whole cell lysates following induction of Myo2p over-expression (upon removal of thiamine, time zero) in *rng3-65* cells. Cells were shifted to the restrictive temperature at 16 hours post-induction, before any appreciable Myo2p expression is detected by antibody (upper blot). The lower blot shows loading controls (Hsp90 levels) for each sample. Protein samples were normalized based on total protein concentration of the lysates. The plot shown below tracks relative levels of Myo2p detected versus time (post-induction). Relative density values for the Myo2p bands were corrected using densities from accompanying Hsp90 bands; final values were normalized whereby 1 is equal to the maximum value. B) Western blots to determine the relative levels of Myo2p produced in the different isolations. Blots were employed (instead of Coomassie staining) to clearly distinguish the Myo2p heavy chain bands in these crude samples. Upper blot: α-Myo2p depicting the relative levels of Myo2p 1-step purified from cells shifted to 37°C (or retained at the permissive growth temperature in accompanying controls) at 16 hours post-induction. The levels shown are from samples harvested at 28 hours post-induction. Samples were diluted 100-fold prior to loading on gels. Lower blot: loading control showing a non-specific band detected by α-Hsp90 antibodies in undiluted samples. Myo2p concentrations were equalized based on the relative levels observed in these blots (prior to testing of the different samples in motility assays; Table 1).

Previous work in yeast and higher eukaryotes has shown that UCS proteins and myosin motors associate with one another [3]–[5], [17], [23], [27], [28]. To further understand the impact of Myo2p’s interaction with Rng3p, we purified myosin motors in the presence and absence of functional Rng3p. Rng3p is essential for cell viability, which precludes isolation of Myo2p from a *rng3*Δ strain. In order to circumvent this, we utilized the temperature-sensitive *rng3-65* mutant. Cells were induced to over-express Myo2p in both *rng3-65* and wild type backgrounds, followed by growth at both permissive and restrictive temperatures. Given the long lag in expression from the *nmt1* promoter, cells were shifted to the restrictive temperature (37°C) 16 hours after induction. This allowed sufficient time to establish an appropriate cell density before attenuating Rng3p function (prior to the start of Myo2p over-expression at ∼20 hours post-induction) (Figure 2A). We included a control experiment where Myo2p over-expression was initiated before attenuating Rng3p function. This was achieved by shifting the cells to the restrictive temperature 22 hours after induction. In all experiments cells were harvested 28 hours post-induction before rapid isolation of Myo2p by one-step purification [23].

In the experiment where cells were shifted 16 hours post-induction, we observed no difference in the rate of Myo2p *in vitro* motility in actin filament gliding assays following its isolation from wild-type cells at 25°C (control) versus those shifted to 37°C (Table 1; Movie S1). Myo2p purified from the *rng3-65* background following growth under permissive conditions (25°C) showed a significantly slower rate of filament gliding (Table 1). More strikingly, the rate of filament gliding was reduced ∼10-fold (Table 1; Movie S1) when Myo2p was expressed and purified from the mutant following the shift to restrictive conditions (37°C). In this case most filaments bound to Myo2p on the cover-slip surface lacked any observable motility. It is worth noting that this form of Myo2p is unstable and prone to aggregation, which has limited further analysis in other types of assays. Our data indicates that Rng3p is required to establish active Myo2p motors.

**Table 1:** Motility rates for Myo2p purified with and without Rng3p function.

Shift time (hours)*		25°C (µm/sec)		37°C (µm/sec)
16	wild-type	0.42±0.12		0.40±0.14
	*rng3-65*	0.30±0.10		0.03±0.02
22	wild-type	0.46±0.07		0.42±0.06
	*rng3-65*	0.34±0.05		0.32±0.06

All values are the mean filament velocity ± SD.

* Shift time is the amount of time following the induction of Myo2p over-expression at which cells were shifted to 37°C to attenuate Rng3p function (in the *rng3-65* strain). Values for wild-type cells are provided as a control. 25°C values represent internal controls that were not shifted to 37°C. At the 16 hour shift Rng3p function is compromised prior to Myo2p over-expression; at the 22 hour shift Rng3p function is compromised following over-expression of Myo2p (see Figure 2A).

The control experiments (where cells were shifted to 37°C at 22 hours post-induction) indicated that Rng3p was not essential for maintaining Myo2p motility once an active population of motors had been synthesized, as previously reported [24]. Myo2p isolated at either temperature from *rng3-65* cells shifted 22 hours post-induction showed no obvious difference in rates of actin filament gliding (Table 1).

While lacking in motility activity, soluble Myo2p was still expressed and isolated from *rng3-65* cells shifted at 16 hours post-induction (Figure 2B). However, the levels of over-expressed Myo2p obtained from *rng3-65* cells were lower than their wild-type counter-parts (Figure 2B). Collectively our findings suggest that Rng3p is required to generate an active and stable population of Myo2p motors.

### A Mutant Form of Myo2p Hyper-sensitive to altered Rng3p Function Lacks Motor Activity

One specific point mutation in the motor domain (*myo2-E1*; G345R) makes Myo2p particularly sensitive to changes in Rng3p function [22]. Unlike other temperature-sensitive *myo2* motor mutants tested, *myo2-E1* exhibits synthetic lethality when combined with temperature-sensitive *rng3* mutants [22]. Furthermore, compared with other motor mutants, *myo2-E1* cells recruit increased levels of Rng3p to the contractile ring [22], [24]. In summary, Myo2p’s reliance on Rng3p and the prevalence of Rng3p-Myo2p associations appear to be exaggerated in *myo2-E1* cells.

**Figure 3:**
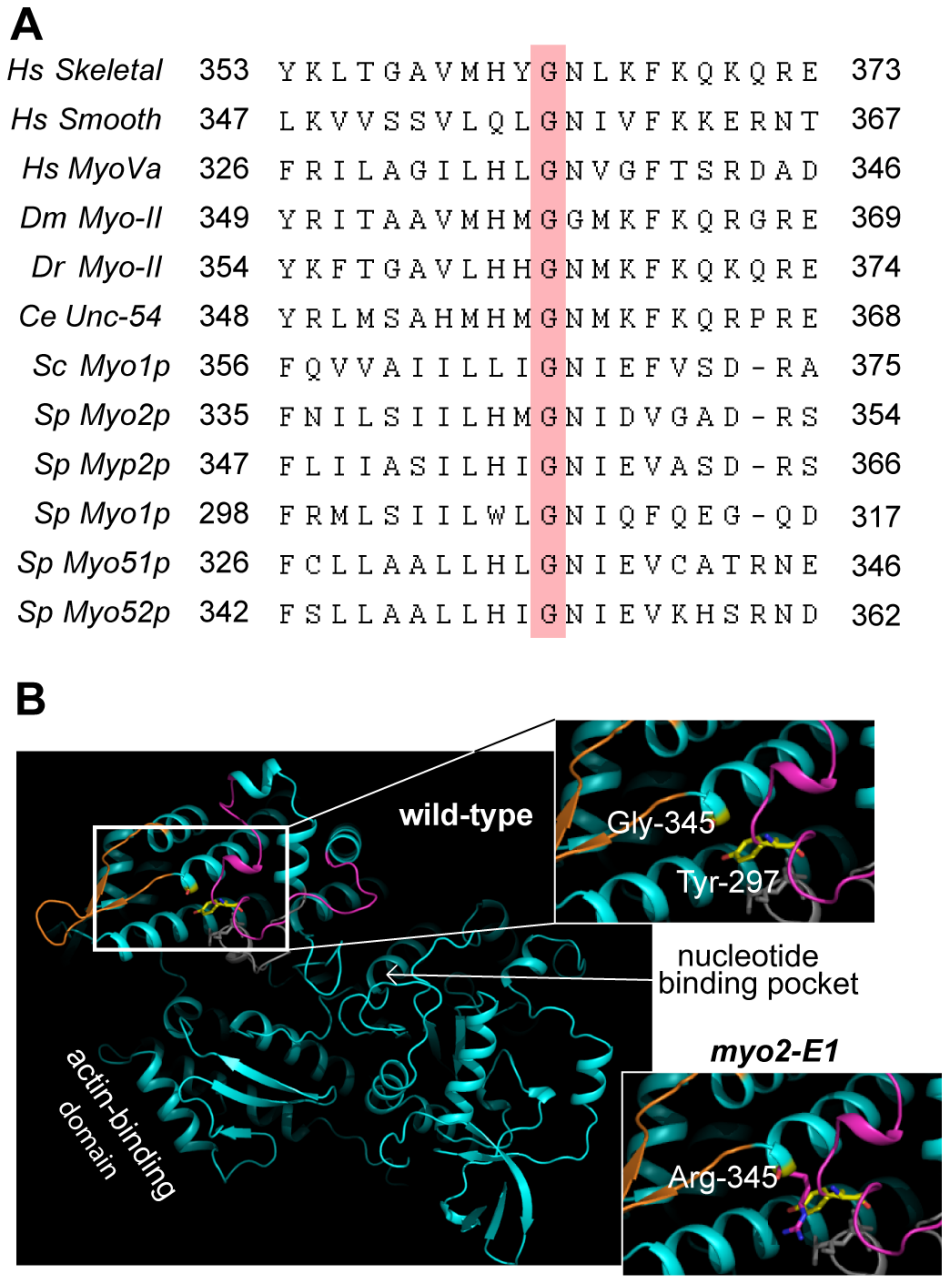
The *myo2-E1* mutation targets a highly conserved residue in the myosin motor domain. A) Amino acid sequence alignment (centered on Gly-345 of Myo2p) of a number of different myosins. The alignment shows that the glycine residue (pink bar) corresponding to the amino acid substitution in *myo2-E1* (G345R) is a highly conserved residue found throughout the myosin super-family. *Hs*: *Homo sapiens*, *Dm*: *Drosophila melanogaster, Dr*: *Danio rerio*, *Ce: Caenorhabditis elegans*, *Sc*: *Saccharomyces cerevisiae*, *Sp*: *Schizosaccharomyces pombe*. Skeletal and smooth refer to muscle myosin-IIs; Unc-54 is a myosin-II from *C. elegans*. B) Homology model of the wild-type Myo2p motor based on the 1br4 crystal structure of smooth muscle myosin-II [53]. Insets highlight the area around the site of the -*E1* mutation (G345R) in wild-type and *myo2-E1*-based structures. The potential introduction of a steric clash between Arg-345 and a conserved tyrosine (Tyr-297) is shown in the *myo2-E1* inset.

We first used homology modeling to examine the structure of a *myo2-E1* motor to gain some insight. The *-E1* mutation is defined by an amino acid substitution (G345R) at a highly conserved glycine (Figure 3A) that is found in every myosin sequence we have so far examined. Modeling of the Gly 345 residue from Myo2p localizes it to the end of a helix near the ATP-binding pocket (Figure 3B). The model suggests that the longer side chain of the Arg 345 in Myo2-E1p introduces a steric clash with a conserved tyrosine (Tyr 297) (Figure 3B), potentially propagating destabilization within the motor domain.

We over-expressed and isolated full-length forms of the wild type and mutant -E1 motors from fission yeast to compare their function *in vitro*. Previous analysis of full-length -E1 in actin filament gliding assays failed to detect any actin binding or motility [23]. Thus, we included methyl-cellulose in our running buffers to favor actin-binding by minimizing diffusion of actin filaments away from motors at the cover-slip surface in the motility chambers. This approach facilitated actin binding in these assays. However, most filaments (99%) bound by -E1 were non-motile, while most filaments bound by wild-type Myo2p were motile (Movie S2). We next assayed the ATPase activity of the motors in the presence of high salt (0.5 M KCl) and the absence of actin. Typical of myosins under such conditions, wild-type motors exhibited no detectable ATPase activity in the presence of MgCl_2_, while showing healthy activity in the presence of CaCl_2_ (Figure S1). However, -E1 motors exhibited relatively low activity under either condition (Figure S1). These experiments performed in the absence of actin suggest that defects in -E1 motors are not specific to actin displacement and motility, and probably reflect a general defect in conformation and function.

**Figure 4:**
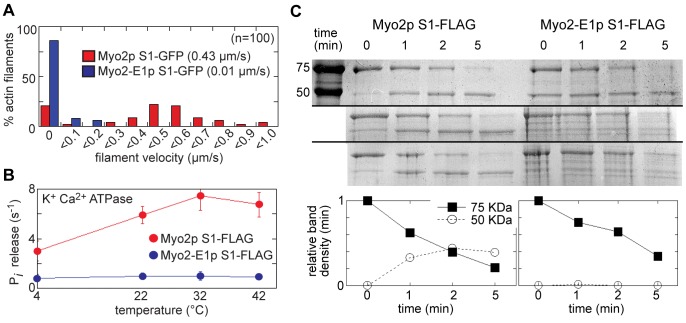
Myo2-E1p motors lack activity and stability. The function of S1 fragments of wild-type Myo2p and -E1 motors were compared in vitro following their over-expression and isolation from fission yeast. A) Histogram comparing the distribution of motility rates of S1-GFP constructs in actin filament gliding assays. Average values are shown inset (0.43±0.05 µm/s for wild-type; 0.01±0.02 µm/s for -E1). B) The ATPase activity of S1-FLAG constructs was assayed and compared over a range of temperatures in the absence of actin and the presence of high salt (0.5 M KCl) with 10 mM CaCl_2_. (n = 3). C) S1-FLAG proteins were subjected to limited proteolysis by trypsin and then run on SDS-PAGE gels. The results of three independent proteolysis experiments are shown. Samples were taken at 0, 1, 2, and 5 minutes following the addition of trypsin before proteolysis was stopped by the addition of SDS-PAGE sample buffer. The far left lane in the top gel shows the running position of 75 and 50 KDa molecular weight standards, bands which run at the same position as the undigested S1 heavy chain band and its primary breakdown product respectively. The intensity of the Coomassie-stained undigested S1 (75 KDa band) and its breakdown product (50 KDa band) were quantified by densitometry. Signals were normalized by setting the intensity of the 75 KDa band to 1.0 and the 50 KDa band to 0 at the 0 min time point. The plot shows the average densitometry values for both the 75 KDa and 50 KDa bands from the three experiments.

The predicted steric clash within the -E1 motor (Figure 3B) may lead to a more labile motor conformation. To examine this further, we over-expressed and purified S1-forms (sub-fragment 1 forms: motor domain plus lever arm and associated light chains) of both wild-type and -E1 motors from fission yeast. The first thing we noted was that yields were typically ∼10-fold lower for S1-E1. Secondly, as with the full-length proteins, we observed similar defects in actin filament gliding (Figure 4A) and high salt ATPase activity (Figure 4B) with the S1-E1 samples. Thirdly, S1-E1 motors were more sensitive to trypsin digestion in limited proteolysis experiments. As shown in Figure 4C, while both S1 heavy chains are degraded at a similar rate, wild-type motors breakdown into a relatively stable lower molecular weight form. However, the lower molecular weight form of -E1 failed to accumulate over time following protease addition (Figure 4C), suggesting an altered conformation more sensitive to proteolysis. In summary, all of our in vitro experiments with full-length and S1 forms of the -E1 protein suggest that this mutant form lacks activity due to defects in stability and activity, defects which presumably dictate increased Rng3p recruitment and regulation in vivo.

We also attempted to correlate our in vitro findings with experiments further examining the inter-dependence of Rng3p and Myo2p function in vivo. We tested whether over-expression of Rng3p could rescue the *myo2-E1* mutant given the increased abundance of Rng3p observed at actomyosin rings in this mutant [22]. Rng3p over-expression did not rescue the *myo2-E1* mutant (Figure S2); likewise over-expression of Myo2p did not rescue the cytokinesis defects of a *rng3-65* mutant (Figure S2). However, such experiments are difficult to interpret because over-expression of either protein has previously been shown to be toxic in wild-type backgrounds [23], [39], [40].

### Rng3p is Specific to Myosin-II

**Figure 5:**
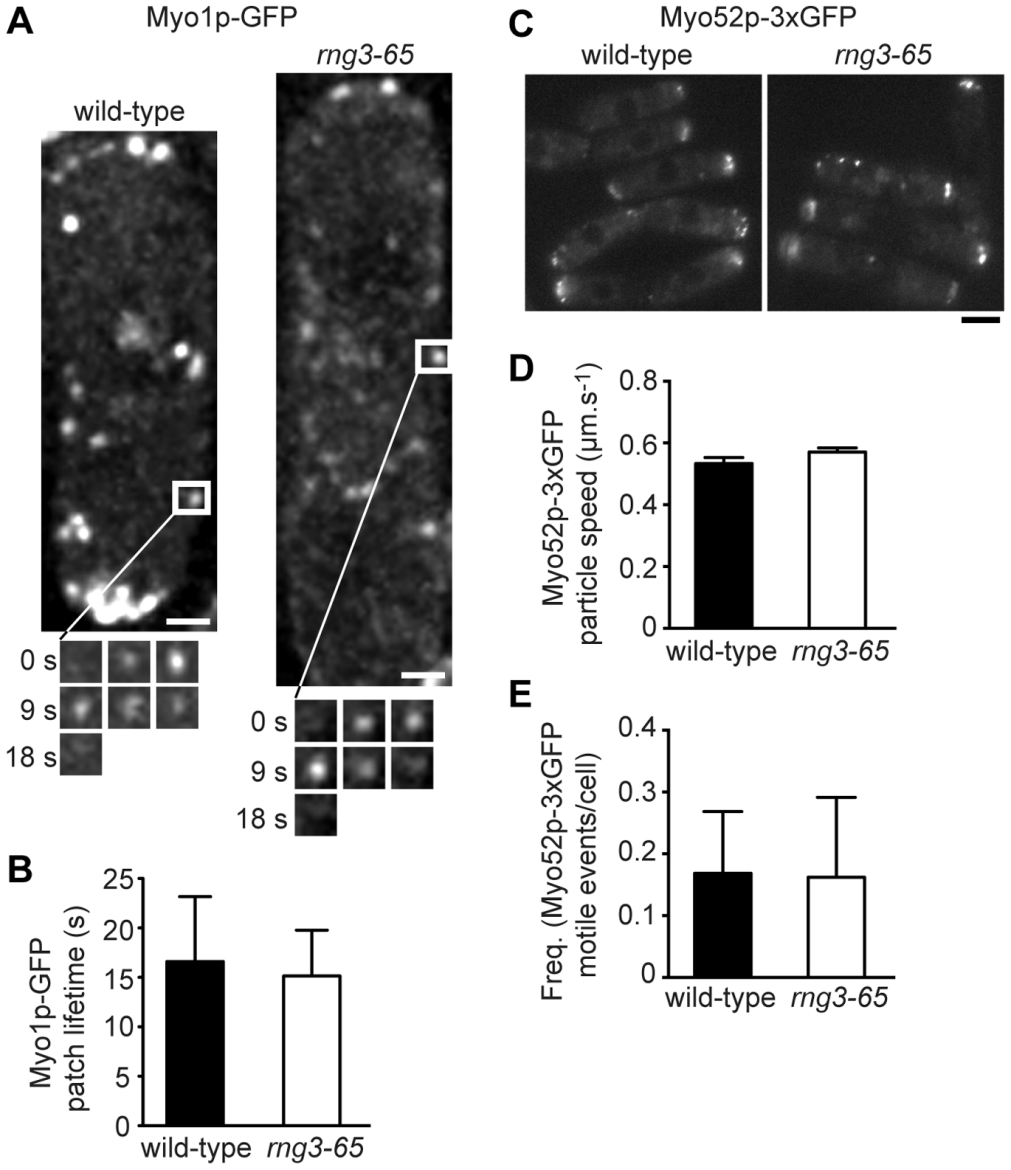
Unconventional fission yeast myosins Myo1p and Myo52p do not require Rng3p for function. Myo1p localization and patch lifetimes (A, B) and Myo52p localization and directed intra-cellular motility (C–E) were assayed. A) Myo1p-GFP patch localization in representative wild-type and *rng3-65* cells is shown following a 5 hour period of growth at 37°C to attenuate Rng3p function in the mutant. White squares in the main images indicate patches whose lifetimes are charted by the time-lapse images shown in the montages below (0–18 s). Bars: 1 µm. B) Average Myo1p patch lifetimes from wild-type and *rng3-65* cells treated as described in A (n = 20). C) Myo52p-3xGFP localization in representative wild-type and *rng3-65* cells is shown following a 5 hour period of growth at 37°C to attenuate Rng3p function in the mutant. Bar: 4 µm. D) Plots showing the average Myo52p-3xGFP particle motility rates in wild-type versus *rng3-65* cells following 5 hours of growth at 37°C (wild-type, n = 239; *rng3-65*, n = 218). E) Frequency of Myo52p-3xGFP particle motility (events/cell/2 min movie) following 5 hours of growth at 37°C (from data shown in D).

*rng3-65* cells show obvious defects in contractile ring formation and cell morphology reflecting a role for Rng3p in Myo2p function during cytokinesis [22]. It was recently proposed that Rng3p works with all the myosins in fission yeast [26]. We turned to live cell imaging of wild-type and *rng3-65* cells to assess myosin-I (Myo1p) and myosin-V (Myo52p) function *in vivo*. We first compared the lifetime of Myo1p at endocytic actin patches. Previous work in budding yeast and fission yeast has shown that defects in myosin-I function lead to an increase in the lifetime of myosin-I at actin patches [41]–[43]. We found that attenuating Rng3p function (by shifting cells to 37°C for 5 hours) had no effect on Myo1p patch dynamics (Movie S3). Myo1p patches showed similar signal intensities and average lifetimes in wild-type and *rng3-65* cells (Figure 5A–B).

We next assayed the type-V myosin Myo52p to see whether its function relied on Rng3p. Myo52p is involved in intracellular transport and can be visualized moving along actin cables within the cell [42], [44]. Firstly, we observed no defects in the intensity or pattern of Myo52p localization in *rng3-65* cells grown at 37°C (Figure 5C). To assess motor function directly, we measured Myo52p particle velocity *in vivo* but found no significant differences in speeds (Figure 5D; Movie S4) or event frequency (Figure 5E) irrespective of whether Rng3p function is perturbed. Taken together, our *in vivo* findings suggest that Rng3p is specific for Myo2p and cytokinesis, and is not required for myosin-I and myosin-V function at other actin structures.

**Figure 6:**
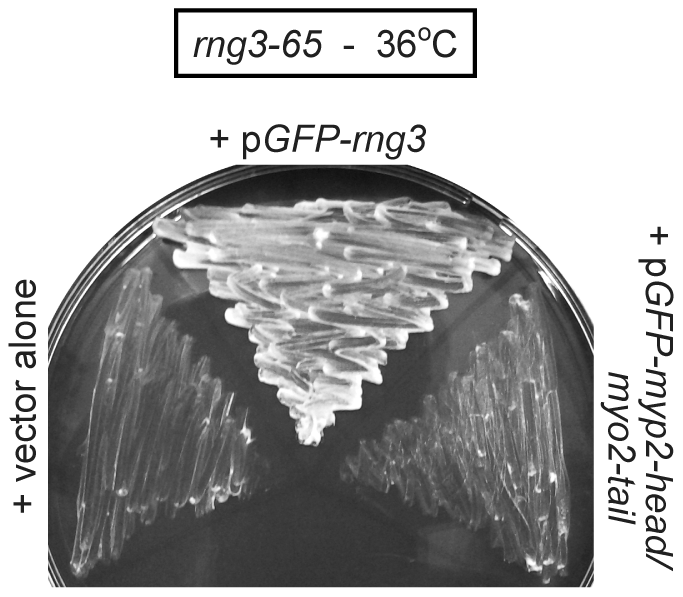
Rng3p is required the function of non-essential myosin-II Myp2p. A Myp2p-head/Myo2p-tail chimera construct was tested for its ability to rescue the growth of temperature-sensitive *rng3-65* mutant cells under restrictive growth conditions (36°C). Plasmid transformants were grown under permissive conditions (25°C) on EMM-Leu^−^ Ura^−^ minimal media plates before re-streaking and subsequent incubation on plates at 36°C. Double drop-out plates were employed to accommodate the differing markers of the p*GFP-myp2-head/myo2-tail* (*LEU2*) and p*GFP-rng3* (*ura4^+^*) plasmids. The vector alone transformant (left, negative control) carried empty *LEU2* and *ura4^+^* plasmids; the Rng3p transformant (center, positive control) carried an empty *LEU2* vector and p*GFP-rng3*; and the Myp2p-head/Myo2p-tail transformant (right) carried p*GFP-myp2-head/myo2-tail* and an empty *ura4^+^* vector.

Use of a chimera construct allowed us to test whether Rng3p is also required for the function of the non-essential fission yeast myosin-II Myp2p (Figure 6). Like Myo2p, Myp2p functions in cytokinesis and localizes to contractile rings [45]–[47]. However, unlike Myo2p, loss of Myp2p does not prevent cytokinesis given *myp2*Δ cells grow normally and only display mild cytokinesis defects under most conditions [45], [47]. It was previously shown that the Myp2p motor of the *myp2*-head/*myo2*-tail chimera rescues loss of Myo2p motor function and cell growth [34]. The fact that Myo2p motor activity is essential for function and cytokinesis [48] tells us that the Myp2p head in the chimera is a functional motor that can work in place of the Myo2p motor. Since Myo2p motor activity relies on Rng3p one would assume that the chimera could bypass the need for Rng3p and rescue the growth of *rng3-65* mutants if Myp2p functioned independently of Rng3p. However, the *myp2*-head/*myo2*-tail chimera failed to restore viability to arrested *rng3-65* cells (Figure 6). This result suggests that Rng3p is also required for Myp2p motor function. We did not test whether Rng3p was required for the function of Myo51p, the myosin-V found at the contractile ring [49], [50]. While we cannot rule out a role in Myo51p function, Rng3p’s critical role likely lies with myosin-II given *myo2* and *rng3* are both essential genes [39], [40], [51], while deletion of the *myo51* gene does not markedly affect cytokinesis [49], [50].

### Rng3p can be Artificially Targeted to Myosin-I Patches in a *myo1-E1* Mutant

While Rng3p is specific to myosin-II in fission yeast, the budding yeast UCS protein functions with myosin-I and myosin-V. We therefore wished to determine whether Rng3p function was specific to the intrinsic properties of Myo2p motors, or whether it is capable of responding to instability or inactivity inherent to any myosin. In order to do this, we artificially destabilized Myo1p by constructing a *myo1-E1* mutant. Figure 3B highlights the conserved glycine residue that defines the *myo2-E1* mutation and we introduced this same mutation into the motor domain of Myo1p (G308R).

**Figure 7:**
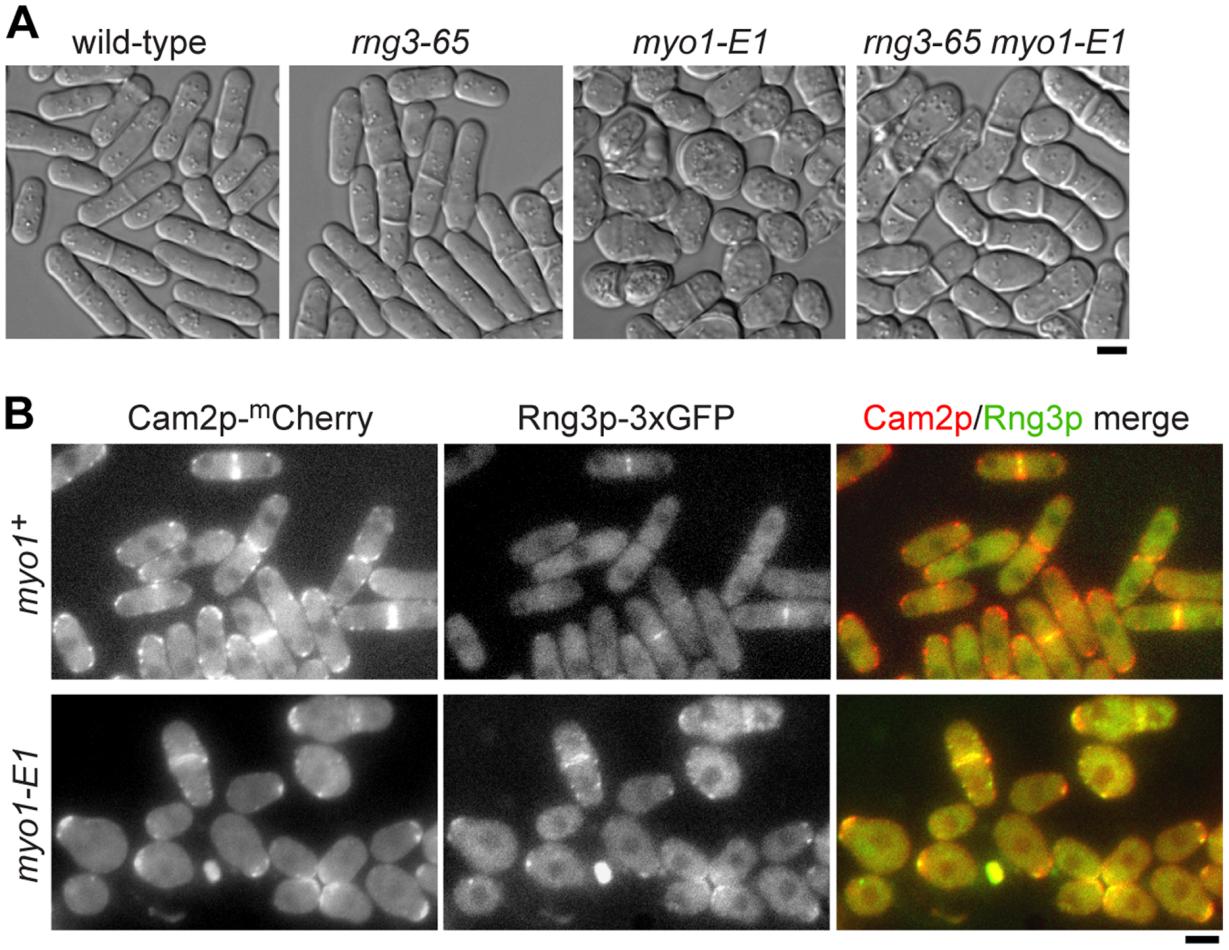
Destabilizing Myo1p motors leads to recruitment of Rng3p to patch structures. The -E1 mutation was introduced into *myo1* (G308R) in the genome to generate the *myo1-E1* strain. A) Representative DIC images of wild-type, *rng3-65*, *myo1-E1*, and *rng3-65 myo1-E1* cells following growth on YE5S media at 25°C. The double mutant was viable and exhibited morphology defects that were a combination of those observed in each of the single mutants (i.e. cells were elongated like *rng3-65* cells and somewhat swollen like *myo1-E1* cells). B) Myo1p (via its light chain Cam2p) and Rng3p localization in wild-type *myo1^+^* (top) and *myo1-E1* mutant (bottom) cells grown in YE5S media at 25°C. *Left*: single plane epi-fluorescence images of Cam2p-^m^Cherry, *center*: single plane epi-fluorescence images of Rng3p-3xGFP, and *right*: merged images of Cam2p (red) and Rng3p (green) signals. Bars: 4 µm.

*myo1-E1* cells appeared rounded (Figure 7A) and grew much slower than wild-type cells (Table 2), morphological and growth defects reminiscent of a *myo1*Δ mutant [31], [52]. Double mutants containing both the *myo1-E1* and *rng3-65* mutations were isolated and exhibited a morphology that was intermediate between the rounded *myo1-E1* phenotype and the elongated *rng3-65* phenotype (Figure 7A). Unlike the synthetic lethality of *myo2-E1 rng3-65* double mutants, any additive phenotype associated with *myo1-E1 rng3-65* cells was not obvious. This presumably reflects the fact that the *myo1-E1* mutant essentially pheno-copied a *myo1* null, and suggests that any role for Rng3p in rescuing the function of Myo1-E1p motors is negligible. That being said, *myo1-E1 rng3-65* double mutants exhibited a slower growth rate than the single mutants (Table 2).

**Table 2:** Growth rates for wild-type, *rng3-65, myo1-E1,* and *rng3-65 myo1-E1* cells.

Strain	Doubling time (min)
wild-type	186±32
*rng3-65*	274±21
*myo1-E1*	341±18
*rng3-65 myo1-E1*	415±45

All values are the mean ± SEM.

Cells were grown at 25°C in YE5S media.

Comparing Myo1p levels in wild-type versus *myo1-E1* cells revealed decreased signal intensity at patches in the mutant (Figure S3). Thus, while Myo1-E1p motors may have some residual function at patches their stability is significantly compromised in vivo. Our previous work indicated that Rng3p localizes faintly to mature contractile rings in wild-type cells [23], and is not detectable with Myo1p at endocytic actin patches (Figure 7B). However, when we visualized Rng3p in *myo1-E1* cells we found that it co-localized with Myo1-E1p in patch-like structures (Figure 7B). Admittedly, complete co-localization at these dynamic patches was impossible to resolve given the exposure times needed to sequentially generate the images (from the relatively faint Rng3p-3xGFP and Cam2p-Cherry signals) versus the short life-times of patches. Nevertheless, it is clear that Rng3p is recruited to patch-like structures in a Myo1-E1p-dependent fashion, a sub-population of which were captured co-localizing with Myo1-E1p patches (Figure 7B). This redistribution in localization indicates that the UCS protein is not strictly exclusive to myosin-II in fission yeast. Our data suggests that UCS proteins are capable of providing general surveillance of myosin motor function which may be unnecessary, minor, or significant depending on the myosin involved or perhaps the requirement of a cellular system to adapt to changes in its environment.

## Discussion

UCS proteins are conserved throughout eukaryotes and have been proposed to act as co-chaperones that work with Hsp90 during the *de novo* folding of myosin motor domains. While lacking the conserved N-terminal Hsp90-binding TPR domain, fungal UCS proteins have been implicated in the function of multiple classes of myosins [22], [26]–[28]. In this study we investigated the mode of action of the fission yeast UCS protein, Rng3p. We propose a chaperone-like role for Rng3p in the regulation of myosin-II motor stability and activity.

### Rng3p Activates Myo2p Motors

We propose that Rng3p stabilizes active motor conformations that support actin filament motility and cytokinesis. Rng3p may also play a role in repairing Myo2p motors that are damaged from forces generated during ring assembly and constriction. This may explain how Rng3p enhances the in vitro motility of purified Myo2p [23]. More notably, our new biochemical data provides a direct link between Rng3p function and Myo2p motor activity. The loss of actin filament gliding when Myo2p motors are generated in the absence of functional Rng3p and the associated drop in yield highlight the importance of Rng3p in Myo2p activity and stability. Similarly, destabilized Myo2p (Myo2-E1p) motors that show an increased dependency on Rng3p function in vivo [22], bound actin without supporting ATPase or motility activity, exhibited an altered conformation, and showed a reduced yield following over-expression and purification from fission yeast. Taken together our findings indicate that Rng3p is the key regulator in establishing Myo2p motor activity.

### Rng3p is Specific to Myosin-II and the Actomyosin Ring

Previous work revealed that Rng3p associates co-translationally with all five of the fission yeast myosins, suggesting that Rng3p functions in the general folding of these myosins [26]. We examined the importance of Rng3p in the function of other myosins that operate throughout the cell cycle at other actin structures. Loss of Rng3p function had no effect on the localization and dynamics of Myo1p or Myo52p. Thus, while Rng3p may be capable of interacting with these unconventional myosins, it is not necessary for their function in the cell. Rng3p function appears to have evolved to establish and maintain Myo2p activity during actomyosin ring assembly and constriction at cytokinesis. Rng3p was also found to be critical for the non-essential myosin-II Myp2p which is also found at the ring. Destabilizing Myo1p via the *myo1-E1* mutation presumably strengthens any transient interactions between this motor and Rng3p, leading to aberrant localization of Rng3p to Myo1p patches where it is not normally found or needed. The ability of Rng3p to associate co-translationally with Myo1p (and its targeting to Myo1-E1p patches in cells) suggests that the UCS protein is not strictly exclusive to myosin-II. Rather, Rng3p probably responds to the intrinsic properties of all myosin classes and can provide surveillance (and regulation) depending on the health or requirement of the motor.

The budding yeast UCS protein (She4p) appears to function somewhat differently to Rng3p. As opposed to working with conventional myosin, She4p is required for unconventional myosin (myosin-I and myosin-V) function [27], [28]. Furthermore, our previous work indicated that while She4p was not required for myosin-I activity, it was required to maintain myosin-I levels in the cell [24]. This may reflect another fundamental difference between the roles of budding and fission yeast UCS proteins: She4p is required to maintain the steady state levels of motors in the cell, whereas Rng3p functions to establish both the activity and levels of motors.

### An Alternative Role for UCS Proteins Besides Functioning as Hsp90 Co-chaperones?

UCS proteins are thought to act as co-chaperones that link unfolded myosin motors bound at their UCS domains to Hsp90 [1], [2], [4]. UCS proteins interact with Hsp90 through their TPR domain [1], [3], yet the fungal UCS proteins lack this domain. While previous work in fission yeast has provided a link between Hsp90, Rng3p, and Myo2p function during cytokinesis [25], UCS proteins can chaperone myosin motors in the absence of Hsp90 [3], [16], [17]. Hsp90 may function upstream of Rng3p in the *de novo* folding of Myo2p motors. In this case, a defect in Hsp90 function may compromise the stability of Myo2p motors, much the same way the *myo2-E1* mutant does. Alternatively, Rng3p and Hsp90 may act at the same time, functioning as independent chaperones that establish and maintain active motor conformations.

Our work does not argue against a role for the UCS protein as a Hsp90 co-chaperone. After all, UCS proteins from higher eukaryotes possess a TPR domain. How this TPR domain influences Hsp90 and myosin motor function may vary depending on the UCS protein, the myosin substrate, or cellular process. While the UCS protein can participate in the Hsp90-dependent folding of muscle myosin-II motors [4], [5], the *C. elegans* UCS protein works antagonistically to Hsp90 as the two proteins compete for myosin-II binding [15]. Our work from fission yeast, combined with recent findings showing that the *C. elegans* UCS domain alone can support full function in vivo [15] suggest an additional role for UCS proteins independent of Hsp90 and *de novo* folding.

## Supporting Information

Figure S1 **Comparing the ATPase activity of Myo2p and Myo2-E1p.** The ATPase activity of full-length Myo2p and Myo2-E1p was assayed in the absence of actin and the presence of high salt (0.5 M KCl) with 10 mM MgCl_2_or 10 mM CaCl_2_.
(TIF)

Figure S2 **Over-expression of Rng3p or Myo2p does not rescue the lethality of** ***myo2-E1*** **or** ***rng3-65*** **mutants.** A) Temperature-sensitive *myo2-E1* cells were transformed with pDS573a-*LEU2* (empty vector control), pYFP*-myo2* (positive control expressing YFP-Myo2p from the *myo2* promoter), and pGST*-rng3-FL* (over-expression construct expressing GST-Rng3p from the high-strength *3xnmt1* inducible promoter). Transformants were isolated on EMM-Leu^−^ (+ thiamine) plates at 25°C. *Left*: cells were re-streaked onto EMM-Leu^−^ plates (lacking thiamine) and grown at 36°C to induce over-expression of Rng3p and attenuate Myo2-E1p function. B) Temperature-sensitive *rng3-65* cells were transformed with pDS573a (empty vector control) and pGFP*-rng3* (positive control expressing GFP-Rng3p from the low-strength *81xnmt1* inducible promoter). A *rng3-65* strain carrying an integrated medium-strength *41xnmt1* inducible promoter (in place of the *myo2* promoter) and pGST-*rlc1* was included to over-express Myo2p. Transformants were isolated on EMM-Ura^−^ (+thiamine) plates at 25°C. *Left*: cells were re-streaked onto EMM-Ura^−^ plates lacking thiamine and grown at 36°C to induce over-expression of Myo2p and attenuate Rng3-65p function. Images on *right*: representative cells from the two plates imaged by DIC microscopy. Bars: 4 µm.
(TIF)

Figure S3 **Myo1p levels are reduced in the** ***myo1-E1*** **mutant.** Merged GFP and CFP fluorescence image of a mixed population of wild-type *myo1-GFP* (with *sad1-CFP*) and mutant *myo1-E1-GFP* cells. Sad1p-CFP was colored in red to distinguish wild-type cells from the *myo1-E1* cells (marked with asterisks). Bar: 4 µm.
(TIF)

Table S1 ***S. pombe*** **strains used in this study.**
(DOC)

Movie S1 **Actin-filament gliding rate is decreased when full-length Myo2p motors are purified in the absence of Rng3p function.** Myo2p motors were expressed and purified from either wild-type or temperature-sensitive *rng3-65* fission yeast cells following 12 hours of growth at the restrictive temperature (37°C). Motors were assayed in vitro in actin filament gliding assays. Movie images captured every 2s and played at 10 frames/s (sped up 20×).
(MOV)

Movie S2 **Mutant Myo2-E1p motors bind actin filaments but do not support filament gliding.** Full-length Myo2p and Myo2-E1p motors were overexpressed and purified from fission yeast cells and tested in actin filament gliding assays. Movie images captured every 2s and played at 10 frames/s (sped up 20×).
(MOV)

Movie S3 **Myo1p patch dynamics are not altered when Rng3p function is perturbed.** Myo1p-GFP patch dynamics in wild-type and *rng3-65* cells grown under restrictive conditions (37°C for 5 hours) to perturb Rng3p function. Movies were generated from maximum projection images (7 z-sections over the depth of the cell) collected every 3s. Movie played at 10 frames/s (sped up 30×).
(MOV)

Movie S4 **Myo52p particle motility is still observed when Rng3p function is perturbed.** Myo52p-3xGFP motile particles in wild-type and *rng3-65* cells grown under restrictive conditions (37°C for 5 hours) to perturb Rng3p function. Movies were generated from single plane images captured every 1.5s. Movie played at 10 frames/s (sped up 15×).
(MOV)

## References

[1] Hutagalung AH, Landsverk ML, Price MG, Epstein HF (2002) The UCS family of myosin chaperones. J Cell Sci 115: 3983–3990. 12356904 10.1242/jcs.00107

[2] Gazda L, Pokrzywa W, Hellerschmied D, Lowe T, Forne I, et al (2013) The myosin chaperone UNC-45 is organized in tandem modules to support myofilament formation in C. elegans. Cell 152: 183–195. 23332754 10.1016/j.cell.2012.12.025 PMC3549490

[3] Barral JM, Hutagalung AH, Brinker A, Hartl FU, Epstein HF (2002) Role of the myosin assembly protein UNC-45 as a molecular chaperone for myosin. Science 295: 669–671. 11809970 10.1126/science.1066648

[4] Liu L, Srikakulam R, Winkelmann DA (2008) Unc45 activates Hsp90-dependent folding of the myosin motor domain. J Biol Chem 283: 13185–13193. 18326487 10.1074/jbc.M800757200 PMC2442312

[5] Srikakulam R, Liu L, Winkelmann DA (2008) Unc45b forms a cytosolic complex with Hsp90 and targets the unfolded myosin motor domain. PLoS One 3: e2137. 18478096 10.1371/journal.pone.0002137 PMC2377097

[6] Anderson MJ, Pham VN, Vogel AM, Weinstein BM, Roman BL (2008) Loss of unc45a precipitates arteriovenous shunting in the aortic arches. Dev Biol 318: 258–267. 18462713 10.1016/j.ydbio.2008.03.022 PMC2483962

[7] Bazzaro M, Santillan A, Lin Z, Tang T, Lee MK, et al.. (2007) Myosin II Co-Chaperone General Cell UNC-45 Overexpression Is Associated with Ovarian Cancer, Rapid Proliferation, and Motility. Am J Pathol. 10.2353/ajpath.2007.070325 PMC2043524 17872978

[8] Guo W, Chen D, Fan Z, Epstein HF (2011) Differential turnover of myosin chaperone UNC-45A isoforms increases in metastatic human breast cancer. J Mol Biol 412: 365–378. 21802425 10.1016/j.jmb.2011.07.012

[9] Melkani GC, Bodmer R, Ocorr K, Bernstein SI (2011) The UNC-45 chaperone is critical for establishing myosin-based myofibrillar organization and cardiac contractility in the Drosophila heart model. PLoS One 6: e22579. 21799905 10.1371/journal.pone.0022579 PMC3143160

[10] Barral JM, Bauer CC, Ortiz I, Epstein HF (1998) Unc-45 mutations in Caenorhabditis elegans implicate a CRO1/She4p-like domain in myosin assembly. J Cell Biol 143: 1215–1225. 9832550 10.1083/jcb.143.5.1215 PMC2133068

[11] Landsverk ML, Li S, Hutagalung AH, Najafov A, Hoppe T, et al (2007) The UNC-45 chaperone mediates sarcomere assembly through myosin degradation in Caenorhabditis elegans. J Cell Biol 177: 205–210. 17438072 10.1083/jcb.200607084 PMC2064129

[12] Bernick EP, Zhang PJ, Du S (2010) Knockdown and overexpression of Unc-45b result in defective myofibril organization in skeletal muscles of zebrafish embryos. BMC Cell Biol 11: 70. 20849610 10.1186/1471-2121-11-70 PMC2954953

[13] Lee CF, Melkani GC, Yu Q, Suggs JA, Kronert WA, et al (2011) Drosophila UNC-45 accumulates in embryonic blastoderm and in muscles, and is essential for muscle myosin stability. J Cell Sci 124: 699–705. 21285246 10.1242/jcs.078964 PMC3039016

[14] Wohlgemuth SL, Crawford BD, Pilgrim DB (2007) The myosin co-chaperone UNC-45 is required for skeletal and cardiac muscle function in zebrafish. Dev Biol 303: 483–492. 17189627 10.1016/j.ydbio.2006.11.027

[15] Ni W, Hutagalung AH, Li S, Epstein HF (2011) The myosin-binding UCS domain but not the Hsp90-binding TPR domain of the UNC-45 chaperone is essential for function in Caenorhabditis elegans. J Cell Sci 124: 3164–3173. 21914819 10.1242/jcs.087320 PMC3706032

[16] Kaiser CM, Bujalowski PJ, Ma L, Anderson J, Epstein HF, et al (2012) Tracking UNC-45 chaperone-myosin interaction with a titin mechanical reporter. Biophys J 102: 2212–2219. 22824286 10.1016/j.bpj.2012.03.013 PMC3341559

[17] Melkani GC, Lee CF, Cammarato A, Bernstein SI (2010) Drosophila UNC-45 prevents heat-induced aggregation of skeletal muscle myosin and facilitates refolding of citrate synthase. Biochem Biophys Res Commun 396: 317–322. 20403336 10.1016/j.bbrc.2010.04.090 PMC2888609

[18] Goyal A, Takaine M, Simanis V, Nakano K (2011) Dividing the spoils of growth and the cell cycle: The fission yeast as a model for the study of cytokinesis. Cytoskeleton (Hoboken) 68: 69–88. 21246752 10.1002/cm.20500 PMC3044818

[19] Pollard TD, Wu JQ (2010) Understanding cytokinesis: lessons from fission yeast. Nat Rev Mol Cell Biol 11: 149–155. 20094054 10.1038/nrm2834 PMC2819279

[20] Coffman VC, Nile AH, Lee IJ, Liu H, Wu JQ (2009) Roles of formin nodes and myosin motor activity in Mid1p-dependent contractile-ring assembly during fission yeast cytokinesis. Mol Biol Cell 20: 5195–5210. 19864459 10.1091/mbc.E09-05-0428 PMC2793295

[21] Stark BC, Sladewski TE, Pollard LW, Lord M (2010) Tropomyosin and myosin-II cellular levels promote actomyosin ring assembly in fission yeast. Mol Biol Cell 21: 989–1000. 20110347 10.1091/mbc.E09-10-0852 PMC2836979

[22] Wong KC, Naqvi NI, Iino Y, Yamamoto M, Balasubramanian MK (2000) Fission yeast Rng3p: an UCS-domain protein that mediates myosin II assembly during cytokinesis. J Cell Sci 113 (Pt 13): 2421–2432. 10.1242/jcs.113.13.2421 10852821

[23] Lord M, Pollard TD (2004) UCS protein Rng3p activates actin filament gliding by fission yeast myosin-II. J Cell Biol 167: 315–325. 15504913 10.1083/jcb.200404045 PMC2172548

[24] Lord M, Sladewski TE, Pollard TD (2008) Yeast UCS proteins promote actomyosin interactions and limit myosin turnover in cells. Proc Natl Acad Sci U S A 105: 8014–8019. 18523008 10.1073/pnas.0802874105 PMC2430351

[25] Mishra M, D'Souza V M, Chang KC, Huang Y, Balasubramanian MK (2005) Hsp90 protein in fission yeast Swo1p and UCS protein Rng3p facilitate myosin II assembly and function. Eukaryot Cell 4: 567–576. 15755919 10.1128/EC.4.3.567-576.2005 PMC1087793

[26] Amorim MJ, Mata J (2009) Rng3, a member of the UCS family of myosin co-chaperones, associates with myosin heavy chains cotranslationally. EMBO Rep 10: 186–191. 19098712 10.1038/embor.2008.228 PMC2637312

[27] Toi H, Fujimura-Kamada K, Irie K, Takai Y, Todo S, et al (2003) She4p/Dim1p interacts with the motor domain of unconventional myosins in the budding yeast, Saccharomyces cerevisiae. Mol Biol Cell 14: 2237–2249. 12808026 10.1091/mbc.E02-09-0616 PMC194874

[28] Wesche S, Arnold M, Jansen RP (2003) The UCS domain protein She4p binds to myosin motor domains and is essential for class I and class V myosin function. Curr Biol 13: 715–724. 12725728 10.1016/s0960-9822(03)00264-1

[29] Bahler J, Wu JQ, Longtine MS, Shah NG, McKenzie A 3rd, et al (1998) Heterologous modules for efficient and versatile PCR-based gene targeting in Schizosaccharomyces pombe. Yeast 14: 943–951. 9717240 10.1002/(SICI)1097-0061(199807)14:10<943::AID-YEA292>3.0.CO;2-Y

[30] Moreno S, Klar A, Nurse P (1991) Molecular genetic analysis of fission yeast Schizosaccharomyces pombe. Methods Enzymol 194: 795–823. 2005825 10.1016/0076-6879(91)94059-l

[31] Lee WL, Bezanilla M, Pollard TD (2000) Fission yeast myosin-I, Myo1p, stimulates actin assembly by Arp2/3 complex and shares functions with WASp. J Cell Biol 151: 789–800. 11076964 10.1083/jcb.151.4.789 PMC2169449

[32] Sladewski TE, Previs MJ, Lord M (2009) Regulation of fission yeast myosin-II function and contractile ring dynamics by regulatory light-chain and heavy-chain phosphorylation. Mol Biol Cell 20: 3941–3952. 19570908 10.1091/mbc.E09-04-0346 PMC2735492

[33] Ho SN, Hunt HD, Horton RM, Pullen JK, Pease LR (1989) Site-directed mutagenesis by overlap extension using the polymerase chain reaction. Gene 77: 51–59. 2744487 10.1016/0378-1119(89)90358-2

[34] Bezanilla M, Pollard TD (2000) Myosin-II tails confer unique functions in Schizosaccharomyces pombe: characterization of a novel myosin-II tail. Mol Biol Cell 11: 79–91. 10637292 10.1091/mbc.11.1.79 PMC14758

[35] Kron SJ, Spudich JA (1986) Fluorescent actin filaments move on myosin fixed to a glass surface. Proc Natl Acad Sci U S A 83: 6272–6276. 3462694 10.1073/pnas.83.17.6272 PMC386485

[36] Spudich JA, Watt S (1971) The regulation of rabbit skeletal muscle contraction. I. Biochemical studies of the interaction of the tropomyosin-troponin complex with actin and the proteolytic fragments of myosin. J Biol Chem 246: 4866–4871. 4254541

[37] Reck-Peterson SL, Tyska MJ, Novick PJ, Mooseker MS (2001) The yeast class V myosins, Myo2p and Myo4p, are nonprocessive actin-based motors. J Cell Biol 153: 1121–1126. 11381095 10.1083/jcb.153.5.1121 PMC2174330

[38] Henkel RD, VandeBerg JL, Walsh RA (1988) A microassay for ATPase. Anal Biochem 169: 312–318. 2968057 10.1016/0003-2697(88)90290-4

[39] Kitayama C, Sugimoto A, Yamamoto M (1997) Type II myosin heavy chain encoded by the myo2 gene composes the contractile ring during cytokinesis in Schizosaccharomyces pombe. J Cell Biol 137: 1309–1319. 9182664 10.1083/jcb.137.6.1309 PMC2132538

[40] May KM, Watts FZ, Jones N, Hyams JS (1997) Type II myosin involved in cytokinesis in the fission yeast, Schizosaccharomyces pombe. Cell Motil Cytoskeleton 38: 385–396. 9415380 10.1002/(SICI)1097-0169(1997)38:4<385::AID-CM8>3.0.CO;2-2

[41] Attanapola SL, Alexander CJ, Mulvihill DP (2009) Ste20-kinase-dependent TEDS-site phosphorylation modulates the dynamic localisation and endocytic function of the fission yeast class I myosin, Myo1. J Cell Sci 122: 3856–3861. 19808887 10.1242/jcs.053959

[42] Clayton JE, Sammons MR, Stark BC, Hodges AR, Lord M (2010) Differential regulation of unconventional fission yeast myosins via the actin track. Curr Biol 20: 1423–1431. 20705471 10.1016/j.cub.2010.07.026

[43] Sun Y, Martin AC, Drubin DG (2006) Endocytic internalization in budding yeast requires coordinated actin nucleation and myosin motor activity. Dev Cell 11: 33–46. 16824951 10.1016/j.devcel.2006.05.008

[44] Grallert A, Martin-Garcia R, Bagley S, Mulvihill DP (2007) In vivo movement of the type V myosin Myo52 requires dimerisation but is independent of the neck domain. J Cell Sci 120: 4093–4098. 18003699 10.1242/jcs.012468

[45] Bezanilla M, Forsburg SL, Pollard TD (1997) Identification of a second myosin-II in Schizosaccharomyces pombe: Myp2p is conditionally required for cytokinesis. Mol Biol Cell 8: 2693–2705. 9398685 10.1091/mbc.8.12.2693 PMC25737

[46] Bezanilla M, Wilson JM, Pollard TD (2000) Fission yeast myosin-II isoforms assemble into contractile rings at distinct times during mitosis. Curr Biol 10: 397–400. 10753748 10.1016/s0960-9822(00)00420-6

[47] Motegi F, Nakano K, Kitayama C, Yamamoto M, Mabuchi I (1997) Identification of Myo3, a second type-II myosin heavy chain in the fission yeast Schizosaccharomyces pombe. FEBS Lett 420: 161–166. 9459302 10.1016/s0014-5793(97)01510-x

[48] Naqvi NI, Eng K, Gould KL, Balasubramanian MK (1999) Evidence for F-actin-dependent and -independent mechanisms involved in assembly and stability of the medial actomyosin ring in fission yeast. Embo J 18: 854–862. 10022828 10.1093/emboj/18.4.854 PMC1171178

[49] Motegi F, Arai R, Mabuchi I (2001) Identification of two type V myosins in fission yeast, one of which functions in polarized cell growth and moves rapidly in the cell. Mol Biol Cell 12: 1367–1380. 11359928 10.1091/mbc.12.5.1367 PMC34590

[50] Win TZ, Gachet Y, Mulvihill DP, May KM, Hyams JS (2001) Two type V myosins with non-overlapping functions in the fission yeast Schizosaccharomyces pombe: Myo52 is concerned with growth polarity and cytokinesis, Myo51 is a component of the cytokinetic actin ring. J Cell Sci 114: 69–79. 11112691 10.1242/jcs.114.1.69

[51] Balasubramanian MK, Helfman DM, Hemmingsen SM (1992) A new tropomyosin essential for cytokinesis in the fission yeast S. pombe. Nature 360: 84–87. 1436080 10.1038/360084a0

[52] Toya M, Motegi F, Nakano K, Mabuchi I, Yamamoto M (2001) Identification and functional analysis of the gene for type I myosin in fission yeast. Genes Cells 6: 187–199. 11260263 10.1046/j.1365-2443.2001.00414.x

[53] Dominguez R, Freyzon Y, Trybus KM, Cohen C (1998) Crystal structure of a vertebrate smooth muscle myosin motor domain and its complex with the essential light chain: visualization of the pre-power stroke state. Cell 94: 559–571. 9741621 10.1016/s0092-8674(00)81598-6

