# Supplementary figures and images for: UCS Protein Rng3p Is Essential for Myosin-II Motor Activity during Cytokinesis in Fission Yeast
Source: PLoS One. 2013 Nov 14;8(11):e79593. doi: 10.1371/journal.pone.0079593 (PMC3828377; doi:10.1371/journal.pone.0079593)

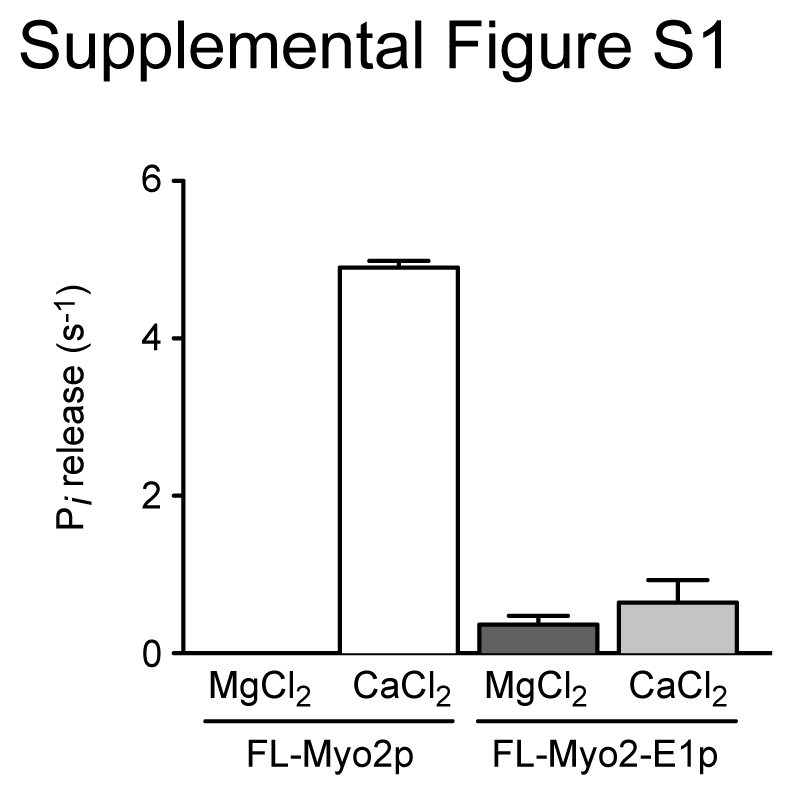

Supplement: Figure S1 — Comparing the ATPase activity of Myo2p and Myo2-E1p. The ATPase activity of full-length Myo2p and Myo2-E1p was assayed in the absence of actin and the presence of high salt (0.5 M KCl) with 10 mM MgCl2or 10 mM CaCl2. (TIF) [file pone.0079593.s001.tif]

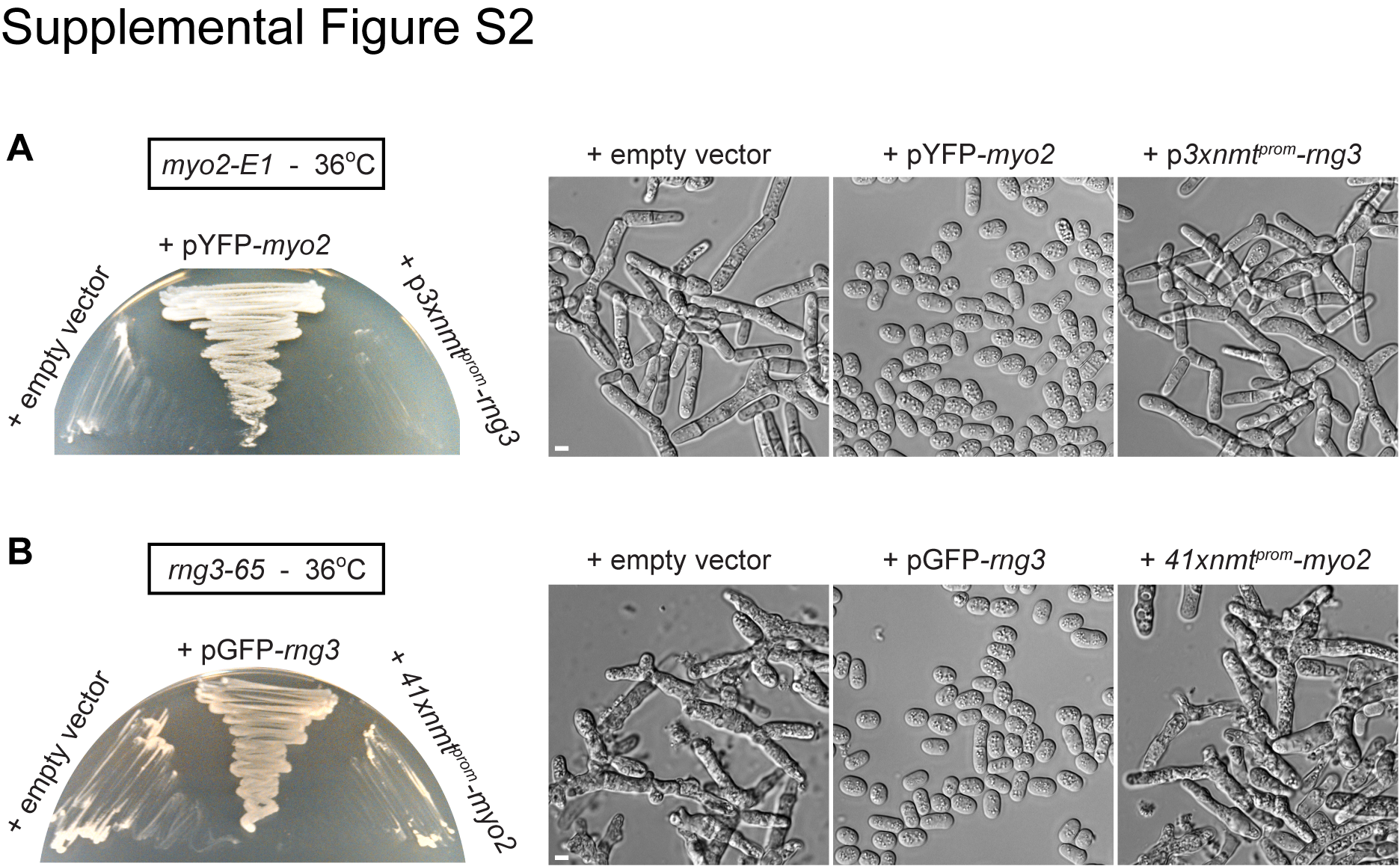

Supplement: Figure S2 — Over-expression of Rng3p or Myo2p does not rescue the lethality of myo2-E1 or rng3-65 mutants. A) Temperature-sensitive myo2-E1 cells were transformed with pDS573a-LEU2 (empty vector control), pYFP-myo2 (positive control expressing YFP-Myo2p from the myo2 promoter), and pGST-rng3-FL (over-expression construct expressing GST-Rng3p from the high-strength 3xnmt1 inducible promoter). Transformants were isolated on EMM-Leu− (+ thiamine) plates at 25°C. Left: cells were re-streaked onto EMM-Leu− plates (lacking thiamine) and grown at 36°C to induce over-expression of Rng3p and attenuate Myo2-E1p function. B) Temperature-sensitive rng3-65 cells were transformed with pDS573a (empty vector control) and pGFP-rng3 (positive control expressing GFP-Rng3p from the low-strength 81xnmt1 inducible promoter). A rng3-65 strain carrying an integrated medium-strength 41xnmt1 inducible promoter (in place of the myo2 promoter) and pGST-rlc1 was included to over-express Myo2p. Transformants were isolated on EMM-Ura− (+thiamine) plates at 25°C. Left: cells were re-streaked onto EMM-Ura− plates lacking thiamine and grown at 36°C to induce over-expression of Myo2p and attenuate Rng3-65p function. Images on right: representative cells from the two plates imaged by DIC microscopy. Bars: 4 µm. (TIF) [file pone.0079593.s002.tif]

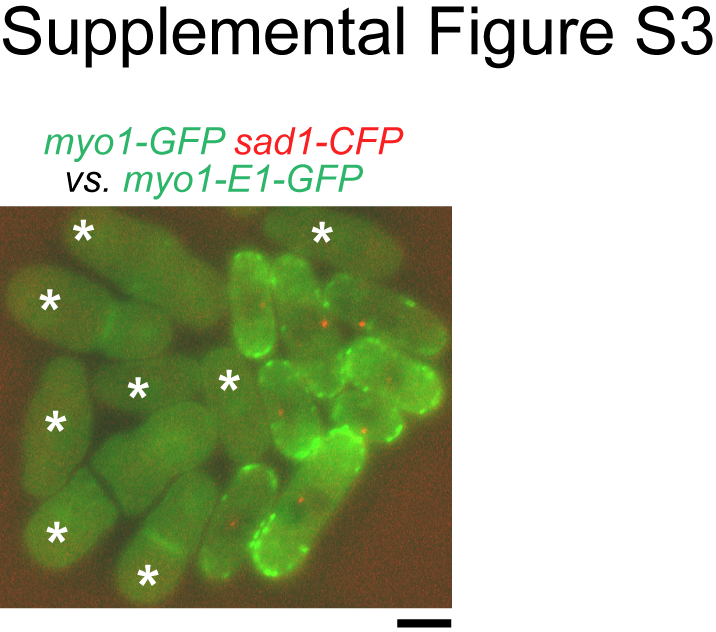

Supplement: Figure S3 — Myo1p levels are reduced in the myo1-E1 mutant. Merged GFP and CFP fluorescence image of a mixed population of wild-type myo1-GFP (with sad1-CFP) and mutant myo1-E1-GFP cells. Sad1p-CFP was colored in red to distinguish wild-type cells from the myo1-E1 cells (marked with asterisks). Bar: 4 µm. (TIF) [file pone.0079593.s003.tif]
