# Supplementary material for: UCS Protein Rng3p Is Essential for Myosin-II Motor Activity during Cytokinesis in Fission Yeast
Source: PLoS One. 2013 Nov 14;8(11):e79593. doi: 10.1371/journal.pone.0079593 (PMC3828377; doi:10.1371/journal.pone.0079593)
Supplement: Table S1 — S. pombe strains used in this study. (DOC) [file pone.0079593.s004.doc]

**Table S1.** *S. pombe* strains used in this study.

| Strain | Genotype | Source |
| --- | --- | --- |
| MLP 479 | *h- leu1-32 ura4∆:kan^R^* |  |
| MLP 509 | *h- leu1-32 ura4∆:kan^R^ nat^R^:nmt41-myo2* |  |
| MLP 586 | *h- leu1-32 ura4∆:kan^R^ his7-366 ade6-M216 nat^R^:nmt41-myo2 rng3-65* |  |
| MLP 469 | *h- leu1-32 ura4-∆18 his7-366 ade6-M216 nat^R^:nmt41-myo2-E1* |  |
| TP 73 | *h- leu1-32 ura4-∆18 his7-366 ade6-M216 myo2-E1* | M. Balasubramanian |
| MLY 422 | *h- leu1-32 myo1-^m^GFP:kan^R^* | This study |
| BS 44 | *h- ade6-M216 leu1-32 his3-∆1 rng3-65*  *myo1-^m^GFP:kan^R^* | This study |
| LP 99 | *h- ade6-M216 leu1-32 his3-∆1 ura4-∆18*  *myo52-3xGFP:kan^R^* | This study |
| BS 56 | *h- ade6-M216 leu1-32 his3-∆1 rng3-65*  *myo52-3xGFP:kan^R^* | This study |
| MLP 586 | *h+ ade6-M216 leu1-32 his7-366 ura4∆:kan^R^ rng3-65* |  |
| BS 74A | *h- ade6-M216 leu1-32 his3-∆1 rng3-65* | This study |
| VS-ml308 | *h- ade6-M216 leu1-32 his3-∆1 ura4-∆18 myo1-E1* | This study |
| BS 74C | *h- ade6-M216 leu1-32 his3-∆1 rng3-65 myo1-E1* | This study |
| MLY 933 | *h- ade6 ura4-∆18 leu1-32 his3-∆1 cam2-^m^Cherry:nat^R^ rng3-3xGFP:kan^R^* | This study |
| MLY 939 | *h+ ade6-M216 ura4-∆18 leu1-32 his3-∆1 myo1-E1*  *cam2-^m^Cherry:nat^R^ rng3-3xGFP:kan^R^* | This study |
| MLY 703 | *h+ ade6 ura4-∆18 leu1-32 his3-∆1 myo1-GFP:kan^R^*  *sad1-CFP:kan^R^* | This study |
| MLY 951 | *h- ade6 leu1-32 his3-∆1 ura4-∆18 myo1-E1-GFP:kan^R^* | This study |

**References**

1. Lord M, Sladewski TE, Pollard TD (2008) Yeast UCS proteins promote actomyosin interactions and limit myosin turnover in cells. Proc Natl Acad Sci U S A 105: 8014-8019.

2. Lord M, Pollard TD (2004) UCS protein Rng3p activates actin filament gliding by fission yeast myosin-II. J Cell Biol 167: 315-325.
